# Assessing the Effects of a Real-Life Contact Intervention on Prejudice Toward LGBT People

**DOI:** 10.1007/s10508-021-02046-0

**Published:** 2021-09-09

**Authors:** Florien M. Cramwinckel, Daan T. Scheepers, Tom F. Wilderjans, Robert-Jan B. de Rooij

**Affiliations:** 1 Department of Interdisciplinary Social Science: Youth Studies, Utrecht University, Utrecht, The Netherlands; 2 Social, Economic and Organizational Psychology, Leiden University, Wassenaarseweg 52, 2333 AK Leiden, The Netherlands; 3 Social, Health, and Organizational Psychology, Utrecht University, Utrecht, The Netherlands; 4 Methodology and Statistics Unit, Institute of Psychology, Leiden University, Leiden, The Netherlands; 5 Research Group of Quantitative Psychology and Individual Differences, Faculty of Psychology and Educational Sciences, Katholieke Universiteit, Leuven, Belgium; 6 Leiden Institute for Brain and Cognition, Leiden, The Netherlands; 7 Online Dialogue, Utrecht, The Netherlands

**Keywords:** LGBT, Contact intervention, Prejudice reduction, Sexual prejudice, Diversity, Sexual orientation

## Abstract

Prejudice against sexual and gender minorities (e.g., LGBT people) is quite prevalent and is harmful. We examined an existing—and often-used—contact intervention in pre-existing groups in an educational setting and assessed its effectiveness in reducing different forms of LGBT negativity. We focused particularly on modern LGBT negativity: a relatively subtle form of prejudice, involving ambivalence, denial, and/or the belief that there is too much attention for LGBT prejudice. We used a mixed design in which condition (experimental vs. control group) was the between-participants factor, which was randomized at the group level, and time (pretest vs. posttest vs. follow-up) was the within-participants factor (*N* = 117). Interventions were video recorded and the behavior of LGBT educators and participants was coded. Participants responded positively to the intervention, especially to the LGBT educator’s “coming-out story.” Exploratory analysis of the video data indicated that the perceived effectiveness of the intervention was higher in groups where participants were more engaged, although caution is necessary in interpreting this finding. The most important measure indicated that modern LGBT negativity decreased in the intervention groups directly after the intervention, but returned to baseline levels one week later. However, in the control condition, modern LGBT negativity had increased over time. Taken together, this suggests that an actual reduction in modern LGBT negativity was short-lived (i.e., the intervention effect disappeared within 7 days).

**Supplementary Information:**

The online version contains supplementary material available at 10.1007/s10508-021-02046-0.

## Introduction

Prejudice and violence against sexual and gender minorities (e.g., people who identify as lesbian, gay, bisexual or transgender [LGBT]) occurs quite often, even in relatively tolerant countries as the Netherlands (Buijs et al., 2011; Cramwinckel et al., 2018; Kuyper, 2015). Apart from the direct negative physical consequences (e.g., being a victim of hate crimes), being the target of prejudice on the basis of one’s (perceived) sexual and/or gender identity is psychologically harmful as it can lead to stress, anxiety, and depression (D’Augelli, 2002; Flenar et al., 2017; Meyer, 2003; Pachankis & Lick, 2018). It is thus important that this prejudice be reduced and that negative behaviors are prevented.

There are several programs in place to target sexual orientation and gender identity prejudice (see, e.g., Bartos et al., 2014; Cramwinckel et al., 2018). However, existing interventions are often not scientifically based, thereby lacking a clear theoretical background, a theory of change, and rigorous empirical testing. As a consequence, it remains largely an open question how effective such interventions actually are. The main goal with the current research was to address these issues by evaluating an existing contact intervention to reduce sexual orientation and gender identity prejudice in an educational context.

### Sexual and Gender Prejudice

Sexual orientation and gender identity prejudice is defined as negative attitudes about certain behaviors, individuals or groups based on their (perceived) sexual orientation, gender identity, role or expression.^1^ As is the case for other types of prejudice, like ethnic prejudice, the face of sexual prejudice has changed over the last decades (e.g., Cramwinckel et al., 2018; Morrison & Morrison, 2003; Twenge et al., 2016). Where old-fashioned prejudice is grounded in moral objections and overtly expressed in hostile attitudes and behaviors (“homosexuality is wrong”), contemporary forms of prejudice are more ambivalent in nature (Morrison & Morrison, 2003). For example, while in most Western societies more people than ever explicitly support LGBT rights (Pew Research Center, 2014; Van Lisdonk, 2018), many people still find it offensive to see same-sex couples kiss in public (Buijs et al., 2011; Kuyper, 2015).

Apart from a sense of ambivalence, another characteristic of contemporary prejudice is that it is often expressed in indirect ways (Massey, 2009). An example is the denial that prejudice or discrimination still occurs or the belief that there is too much attention for it in the media. Such indirectly expressed prejudice has been called “modern prejudice” or, specifically in the LGBT context, “modern homonegativity” (Fry et al., 2020; Morrison & Morrison, 2003, 2011).

Although contemporary forms of prejudice are more ambivalent and indirect, they still have a negative impact on its targets. The ambivalence and indirectness of contemporary bias may result in uncertainty among its targets: Is an awkward interaction due to one’s own behavior and characteristics or is it due to prejudice by the interaction partner? (Barreto & Ellemers, 2015; Van Lisdonk, 2018).

These negative consequences of contemporary prejudice can (partly) explain why even in progressive countries such as the Netherlands, where 90% of the people thinks that gay men and women should be able to live their lives as they wish (Kuyper, 2015), sexual or gender minority group members face more negative outcomes in life, compared to the heterosexual and/or cisgendered majority group. More specifically, compared to Dutch heterosexuals, sexual and gender minorities in the Netherlands have more physical and mental health issues, feel less safe, and have worse employment conditions. This is especially the case for people who identify as transgender (Netherlands Institute for Social Research, 2018). Furthermore, suicide attempts are five to ten times as high for LGBT teenagers than for their heterosexual peers (Kuyper, 2015). These negative consequences call for interventions to combat sexual orientation and gender identity prejudice, also in its contemporary form.

### Prejudice Reduction

Prejudice reduction is an important research area, which is growing rapidly in the last decade (Paluck et al., 2021). Prejudice reduction interventions are most often studied in the area of race and/or ethnicity and less often in the area of sexual orientation and/or gender diversity. An often-used intervention method to reduce sexual and gender prejudice are so-called “contact interventions” that can be implemented in, for example, school or college settings (Bartos et al., 2014; Cotten-Huston & Waite, 1999; Kroneman et al., 2019; Walch et al., 2012). During a contact intervention, participants are brought into contact with sexual and gender minority group members, often in combination with providing information or discussing themes in relation to sexual orientation and gender identity. However, actual contact or imagined contact seems a crucial ingredient in such interventions as just providing information has led to mixed results regarding reducing prejudice (Bezrukova et al., 2016; Deese & Dawson, 2013; Hodson et al., 2013; Walters & Rehma, 2013). For example, one study showed that watching a video about transgender children increased knowledge about transgenderism, compared to a control condition where no video was shown (Walters & Rehma, 2013). However, increasing knowledge does not necessarily mean reducing prejudice (Case & Stewart, 2010), and another study even found that people who watched an informational video about the biological underpinnings of same-sex attractions showed an *increase* in prejudice, compared to people who had not watched these videos (Deese & Dawson, 2013).

Contact interventions are based on contact theory (Allport, 1954; Brown & Hewstone, 2005; Pettigrew, 1998). The central tenet of contact theory is that bringing members of different groups into contact will reduce the prejudice among them. The idea is that contact makes commonalities with out-group members salient, and at the same time reduces inter-group anxiety and threat. In turn, this should change negative stereotypes and lower prejudice. Contact interventions have been applied to many inter-group settings, including those where sexual and gender majority group members (i.e., people who identify as heterosexual and/or are cisgendered) are brought into contact with sexual and gender minority group members (Cotten-Huston & Waite, 1999; Smith et al., 2009; Walch et al., 2012).

A typical example of a contact intervention in the context of transphobia is performed by Walch et al. (2012). In their study, students received a lecture on transgenderism, as well as a panel presentation with four transgender people. Results showed that the panel presentation by transgender people was most effective in reducing transphobia among students. Imagining contact may also be a fruitful method of LGBT prejudice reduction. Turner et al. (2007), for example, demonstrated that attitudes toward homosexuals were improved when male heterosexual participants spent a few minutes imagining a positive interaction with a homosexual man.

The classic meta-analysis on inter-group contact by Pettigrew and Tropp (2006) showed a reliable overall effect of contact on reducing prejudice, with an effect size of *r* = −0.21 (see also Bartos et al., 2014; Smith et al., 2009). Notably, the effect size of the studies focusing on sexual prejudice (*r* = −0.27) was the highest of all types of prejudice examined (e.g., prejudice against ethnic groups, or disabled people) and significantly higher than for those other types of prejudice. A similar finding was obtained in a meta-analysis integrating more recent research (Paluck et al., 2021), which showed a reliable overall effect of face-to-face contact on reducing LGBT prejudice, with an effect size of *d* = 0.22. Again, this effect size was substantially larger than that of studies examining the influence of contact on reducing racial prejudice (*d* = 0.10). Together this suggests that stimulating contact is a particularly fruitful way to reduce prejudice against sexual minorities.

The overall positive effect of inter-group contact in reducing prejudice is especially noteworthy when one considers that under particular circumstances contact can also worsen inter-group relations (Cramwinckel et al., 2018; Felten et al., 2015; Kroneman et al., 2019). For example, engaging in open discussion where participants can also voice negative opinions about sexual and gender minorities can increase—rather than reduce—prejudice (Walker et al., 2015).

The finding that—despite its overall positive effect—inter-group contact may sometimes backfire and increase prejudice suggests that there are certain conditions or moderators that make contact more or less fruitful. Indeed, in the context of contact theory, several of such factors have been described. These so-called “contact conditions” include equal status, common goals, the opportunity for in-depth contact (“acquaintance potential”), and support by authorities (Cook, 1985). Although a meta-analysis by Pettigrew and Tropp (2006) indicated that inter-group contact reduced prejudice even when not all contact conditions were met, these conditions tended to further strengthen the effect of contact per se.

### The Current Study

In the current study, we evaluated an often-used and real-life intervention program among first-year psychology students who participated in existing workgroups. The study consisted of three parts. The first part was a pre-measure (t0: the pretest; see Table S1 in the supplementary materials for an overview of all measures on all time-points), which was administered about a week before the contact session. In the pretest, the students’ sexual and gender prejudice and other variables were measured. The second part was the actual contact intervention (t1: the intervention, followed by the posttest), which was only administered to groups in the experimental condition (groups in the control condition were on a waiting list for the contact intervention). The session itself was videotaped. Directly after the contact intervention, participants filled out a post-measure, assessing again prejudice, an evaluation of the contact intervention, and other variables. The final part was a follow-up measure (t2: the follow-up test), administered about a week after the contact intervention where students’ prejudice and other variables were measured.

As sexual and gender prejudice measures, we included modern LGBT negativity, old-fashioned prejudice, attitudes toward public displays of affection, and attitudes toward gender non-conformity. Because we anticipated relatively low levels of prejudice in the current student sample (Lambert et al., 2006), we expected that the most variance (and hence possible effects) would emerge on the modern LGBT negativity scale, and less on the other scales (particularly the old-fashioned scale), due to possible floor effects. Because there are considerable sex differences in sexual orientation and gender identity prejudice, with men scoring typically higher than women (Herek & McLemore, 2013), in the analyses we controlled for participant’s sex. Stimulus materials, raw data, syntaxes, and other study materials are stored on the Open Science Framework and are available via osf.io/s9zwg.

We were mainly interested in whether the intervention program could successfully reduce LGBT prejudice in the current sample. Furthermore, we were also interested in how participants evaluated the intervention session and how they behaved during the intervention, as these insights may help to shape improvements of the intervention.

## Method

### Participants

A total of 117 students (87 women) participated in exchange for course credits. Most participants were Dutch (109 participants). Participants’ ages varied between 17 and 33 years, and the mean age was 19.56 years (*SD* = 2.14). 109 participants self-identified as heterosexual, one participant identified as homosexual/lesbian, four participants identified as bisexual, and three participants identified as “other” (e.g., pansexual). On average, participants knew ± 5 LGBT’s in person (*SD* = 5.5; range, 0–50), of which they considered ± 2 to be good friends (*SD* = 2.2; range, 0–15). Only four people indicated not to know any LGBT people in person, and about one-third (32.5%) did not have any good friend that was LGBT. Sixteen percent of the participants indicated to be religious.^2^ Participants also indicated on a 7-point scale how important religion was for them (1 = *totally unimportant*; 7 = *very important*); on average, participants scored on the lower end of this scale (*M* = 2.86; *SD* = 2.03).

### Design

We used a mixed (between- and within-subjects) design. The between-subjects factor was condition (control condition vs. experimental condition), which was randomized at the group level using a random number generator. The within-subjects factor was time (pretest, t0 vs. posttest, t1 vs. follow-up, t2). Participants in the control condition were on a “waiting-list” and received the contact intervention several weeks after the experimental groups. Group-randomized designs are unlikely to have adequate power for between-group comparisons without at least 8–10 groups per condition (Murray et al., 2004). Therefore, we aimed to collect a minimum of 16 groups in this study and allocated any additional groups to a related study for which we recruited participants simultaneously. Note that for studying the change in prejudice over time (i.e., within-group comparisons), power is good as three measurements were present for each participant and we had in total about 100 participants spread across the (limited number of) groups. Participants participated in existing workgroups as part of a first-year introduction to psychology course. Out of the 32 groups that were approached, 23 groups agreed to participate (~ 72% participation rate). Eighteen groups were included in the current study. The five remaining groups participated in a subsequent study that was executed after the current study, and that will not be discussed here further. Group size in the current study varied between 3 and 10, with a mean of 6.87 participants (*SD* = 1.80).

Eight groups were randomly assigned to the experimental condition (56 students in total), and eight groups were randomly assigned to the control condition (61 students in total). Two groups that were initially planned to participate in the subsequent study were eventually added to the current sample: For one of these groups, there was no educator and, as a consequence, the participants in this groups did not receive any treatment although they completed the pre-measure and the follow-up measure; this group was then added to the control condition of the current study. The other group did participate in the intervention program, but did not receive the additional treatment that was part of the subsequent study and, as a consequence, did receive exactly the same treatment as the participants in the experimental condition of the current study; this group was added to the experimental condition of the current study. This led to a total of *k* = 18 groups.

### Procedure

Below, we describe what happened during each part of the study; see the Measures section for the specific measures used in the current study and the time-points at which they were collected.

#### T0: Pretest

Participants who registered to participate in the study received a link to an online pretest survey (programmed in Qualtrics). The pretest started with an information screen where participants provided informed consent. After providing informed consent, a variety of measures were assessed (see Table S1 in the supplementary materials), such as several scales measuring sexual and gender prejudice and background variables. Participants could also note down any remarks they had. Hereafter, participants were thanked for their participation in the first part of this study.

#### T1: Intervention + T1 posttest (experimental groups only)

Only the experimental groups participated in the intervention. There were on average 11.7 days (*SD* = 6.5; range, 4.6–22.9 days) between the pretest (T0) and the contact intervention (T1). The intervention was taught by experienced educators of the “COC Mid-Netherlands” who identified as sexual or gender identity minority members themselves (four identified as gay men, three as lesbians, and one woman as bisexual), and discussed their personal experiences as minority members during the intervention.

#### The Intervention

In the current research, we examined an existing intervention that was developed and implemented by the organization “COC Mid-Netherlands” (www.cocmiddennederland.nl).^3^ The contact intervention was led by one or two experienced LGBT educators. The contact intervention was executed in a video laboratory and was videotaped by means of four video cameras that were mounted in the corners of the ceiling. This part lasted 45–60 min. Participants were invited to the laboratory where they met one or two educators. Chairs were aligned in a half circle, facing the front of the room where the educators were seated, next to a flip-over board. Each session consisted of two parts: the introduction part and the interaction part. During the introduction part, participants and the educators introduced themselves, and the rules for the meeting were explained, e.g., that participants could ask all questions they wanted, but that they should do so respectfully. Hereafter, participants were asked to write down or mention their associations regarding sexual and gender diversity and formulate one or more questions they had regarding this topic. The questions and associations were written on the flip-over board and were later used as a guideline for discussion. During this first part, factual information about sexual and gender diversity was also discussed (e.g., what sexual and gender diversity entails, how many people are LGBT + , etc.). The introduction part ended with an educator telling his or her personal coming-out story. This story typically consisted of the educator explaining when and how they found out they were LGBT, and how their friends and family reacted to their coming out. The coming-out stories differed between different educators, as they were based on their personal experiences. One example of a personal coming-out story entailed a homosexual educator discussing how difficult it was for him to come out to his parents. He told the participants how he found himself sitting at his parents’ kitchen table every week with a different excuse, until he finally found the courage to tell his mother that he was gay.

Hereafter, the interaction part started, where participants and educators discussed sexual and gender diversity topics and participants got the chance to ask questions. While the first part of the class was relatively similar in all groups, the second part depended on the input of the participants in the introduction part. For example, in some groups, participants asked more questions about transgender people, while in other groups there were more questions on how same-sex couples could become parents. During the second part, the educators tried to discuss all questions that were raised. To this end, several techniques were used. For example, to let participants experience how difficult it can be to “come out,” the “statements-game” was used, where statements were read out, and participants had to stand up when a statement applied to them personally. These statements were somewhat personal, such as “I have stolen something in the past” or “I want to make my parents proud.” Playing this game let participants experience how difficult it could be to stand up and acknowledge something personal. This experience could be compared to a coming out, where someone has to “stand up” and tell people that he or she is not heterosexual. These techniques were based on “best practices” and were often developed by the educators themselves and (as far as we know) not based on theory or empirically tested before.

Together, the intervention combines some of the “contact conditions” as described by contact theory (Cook, 1985), like acquaintance potential (coming-out-story), common goals (several collective tasks), and support by authorities (the school/university that included the intervention in its curriculum). The contact intervention session ended after 45 min; the experimenter then entered the room and notified the educators that they needed to wrap-up the session.

#### T1: Posttest

Directly after the contact intervention, participants in the experimental condition completed the online posttest (T1; posttest) in individual cubicles or on laptops in the room where the session took place. Participants completed questionnaires measuring their evaluation of the contact intervention, modern LGBT negativity, and additional measures. In order to reduce demand characteristics, it was noted several times that there were no right or wrong answers and that we were interested in participants’ personal opinions. Finally, participants could provide remarks and/or ask questions about the research, were thanked for their participation, and told that they would receive an email one week later with a link to the final questionnaire.

#### T2: Follow-up

One week after the contact intervention, participants received a link to the online follow-up test. On average, there were 7.5 days (*SD* = 0.6; range, 6.8–9.0 days) between the intervention + posttest (T1) and the follow-up test (T2). In the follow-up test, participants completed questionnaires measuring their evaluations of the contact intervention, modern LGBT negativity, and additional measures (see Table S1 in the supplementary materials). Finally, participants in the experimental condition were thanked for their participation and received course credits. Participants in the control condition were thanked for their participation and were informed that they would be taking part in the contact intervention at a designated time in the future. For these participants, the full debriefing followed after their participation in the contact intervention.

### Measures

Except for where indicated otherwise, responses to all self-report items were provided using 7-point Likert scales ranging from 1 (*strongly disagree*) to 7 (*strongly agree*); see Table S1 in the supplementary materials for the exact wording of all items, as well as response options and time-points the items were assessed.

#### Prejudice

Modern LGBT negativity (t0, t1, t2) was assessed with an adapted version of Morrison and Morrison’s (2003, 2011) 12-item modern Homonegativity Scale–Gay men (MHS-G; Cronbach’s α: t0 = 0.82, t1 = 0.86, t2 = 0.86), where “gay men” was replaced in all statements by “lesbians, gays, bisexuals and transgenders.” An example item is “Many lesbians, gays, bisexuals and transgendered people use their sexual orientation so that they can obtain special privileges.” Items could be answered using a slider bar ranging from 0 (*strongly disagree*) to 100 (*strongly agree*).

Old-fashioned prejudice (t0, t1, t2) was assessed with the 10-item Revised Short Version of the Attitudes Towards Lesbians and Gay Men Scale (ATLG-R-S5; Herek, 1997; 5 items about lesbians and 5 items about gay men) (α: T0 = 0.64, t1 = 0.61, t2 = 0.61). An example item was “Sex between two men is just plain wrong.”

Attitudes toward public displays of affection (t0, t1, t2) were assessed with four items (Kuyper, 2015), such as “I think it’s offensive when two women kiss in public” (α: t0 = 0.88, t1 = 0.90, t2 = 0.90).

Attitudes toward gender non-conformity (t0, t1, t2) were assessed with four items (Kuyper, 2015) such as “I do not feel comfortable being around women who look masculine” (α: t0 = 0.85, t1 = 0.89, t2 = 0.83).

#### Evaluation of the Intervention

Participants’ self-reported effectiveness of the contact intervention (t1, t2) was assessed with the statement “The contact program has positively changed my views of LGBT people.” Evaluation of the intervention (t1, t2) was assessed with 3 items (α: t1 = 0.76, t2 = 0.83): “I thought the education class was useful,” “I thought the education class was informative,” and “I thought the contact program was useless” (recoded). Two open-ended questions (t1) assessed which aspects of the contact intervention had made the most positive and least positive impression and why. Participants also graded the intervention (t1, t2) and the educators (t1), on scales ranging from 1 (lowest grade) to 10 (highest grade). Experienced empathy after the intervention (t1) was assessed with a self-developed 8-item scale (α at t1 = 0.73). An example item was: “I could empathize with the educators’ stories.” Feelings of unsafety during the intervention (t1) were assessed with a self-developed 9-item scale (α at t1 = 0.82). An example item was “I was afraid that my opinions would be criticized during the education class.” All these questions were only completed by participants in the experimental condition.

#### Behavior During the Intervention

The behaviors by participants and educators during the intervention were coded by two trained research assistants who were masked to conditions and hypotheses, but aware of the general topic of the study (i.e., testing the effectiveness of an interaction). Seven out of eight videos were coded by both coders. To increase inter-coder reliability, the coding of the first few videos was discussed together with the first author, during which the ratings of the two coders were compared across 5-min intervals, and possible discrepancies were discussed.

Initially, coders tallied how often and how long the following behaviors took place: asking a question, making a comment, mentioning a negative stereotype/prejudice, laughing, positive reinforcement, making a positive remark, nodding, raising one’s hand, talking among themselves, and mentioning a positive stereotype/prejudice. However, scoring and interpretation of these different categories proved to be complex. For example, in many of the videos these behaviors did not, or just a few times, occur. Furthermore, it often happened that participants mentioned examples of behaviors or attitudes that they disagreed with, which were hard to score (i.e., is the comment that “some people think that gay men are effeminate, but I disagree” a negative stereotype, a positive remark, or both?). It also happened that the verbal behavior of the participants did not match their non-verbal behaviors (e.g., saying that one has no problem with LGBT people, but laughing when a stereotype is mentioned). As a consequence, coders sometimes had large discrepancies in their coding of the events (i.e., one coder marked such an event as positive and the other as negative). Because of these difficulties, for the current report, we focus on the percentage of time educators and participants who were actively engaged (e.g., talking and making comments) as this was easy to determine in an objective and reliable way.

#### Background Variables

The following demographic variables were assessed: sex, age, sexual orientation, ethnicity (t0), and religiosity (t2). Contact with LGBTs (t0) was assessed with two open-ended questions, assessing how many lesbians, gay men, bisexuals, and/or transgender people participants knew personally, and how many of them they considered to be (good) friends. Finally, to examine the possible role of demand characteristics in reporting (low levels of) prejudice, we included the Social Desirability Scale (Strahan & Gerbasi, 1972, 20 items, α = 0.74) at t0. Answers could be given on a binary scale (true vs. false). An example item was “I'm always willing to admit it when I make a mistake.”

### Analyses Plan

As we were primarily interested in the effect of the intervention on modern LGBT negativity, we first investigated—by means of multilevel modeling—whether modern LGBT negativity could be predicted based on condition and time of measurement (controlling for gender, age, and group size). Next, we also studied the pattern of change over time for the other forms of prejudice (old-fashioned prejudice, attitudes toward gender non-conformity, and attitudes toward public displays of affection).

Two models were built for modern LGBT negativity. First, we built a multilevel model for the experimental condition only, to compare modern LGBT negativity across all three measurements (Model 1: t0, t1, t2). This allowed us to examine the immediate effect of the intervention (t0-t1) and the extent to which this effect lasted over time (t1-t2 and t0 vs t2). Second, we built a multilevel model to compare differences between pre- and follow-up measures of LGBT negativity (t0- t2) between the experimental and control condition (Model 2; note that there are no measurements for the control condition at t1).

For both models, we primarily examined changes in prejudice associated with the intervention. Additionally, we examined whether the intervention effect was influenced by the control variables of sex, age, and group size. We used multilevel modeling in order to deal with the hierarchical structure of the data (i.e., three-level data with repeated measures of prejudice within participants and multiple participants per group, which caused prejudice scores within participants and within groups to be correlated) and examined the within-participant and within-group differences (Singer & Willett, 2003). To this end, we adopted the statistical software R version 3.3.1 and used the "lmer"-function from the "lme4" package. We obtained *p*-values by the Satterthwaite approximation using the "lmerTest" package.

#### Building the Multilevel Model

We constructed multilevel models in the following stepwise fashion: We started with a simple model and added or removed effects until we reached a final model that adequately described our data. In particular, first, an unconditional means model with only random intercepts for participants (Level 2) and groups (Level 3) was fit in order to inspect how the dependent variable varied across time-points, participants, and groups (Step 1). In a second step, we added time as a fixed effect to compare modern LGBT negativity across the three time-points (Step 2). Next, we made the effect of time random across participants (Step 3) and groups (Step 4) in order to examine variations in the slopes for time across participants and groups.

In a next step, we added (Level 2) participant characteristics (i.e., sex and age) as fixed effects to explain between-participant differences in modern LGBT negativity at the beginning of the study and between-participant differences in the (short- and long-term) intervention effect (Step 5). In a subsequent step, we tested whether the between-participant differences from the previous model differed across groups (i.e., random effects at Level 3) and we added (Level 3) group characteristics (i.e., condition and group size) as fixed effects to explain these between-group differences (Step 6). Next, we also added the interaction effects of all fixed effects already in the model (Step 7). Finally, we made the model more parsimonious by eliminating all variables and interaction effects of which deleting them did not substantially decrease model fit, starting with the highest order interaction effects. In each step, we tested with a likelihood-ratio test (LRT) whether adding an effect(s) significantly improved the model fit and whether removing (an) effect(s) did not decrease the model fit significantly.

#### Assumptions and Bootstrap

We tested the multilevel model assumptions for the final models. In particular, we tested for linearity, normality, and homoscedasticity of the residuals. Unless described otherwise, we found no clear violations of these assumptions. To determine the robustness of our conclusions against possible model assumption violations, we also performed a clustered bootstrap analysis with 10.000 bootstrap samples (Davidson & Hinkley, 1997; Deen & de Rooij, 2020) and compared our obtained final model with the bootstrap results.

## Results

### Attrition

Of the 117 participants, 10 participants completed part of this research but their data were excluded from the analyses for one or more of the following reasons: (1) because they did not take part in the contact intervention while they were allocated to the experimental condition or because they took part in the contact intervention while they were allocated to the control condition, (2) they did not complete the post-test and follow-up measures, and/or (3) they did not provide informed consent or withdrew consent verbally. As such, data from 107 participants were included in the multilevel analyses: 104 participants had complete modern LGBT negativity data on all three time-points; for two participants, there were missing data at t0, and for one participant there were missing data at both t1 and t2. However, an advantage of multilevel analysis is that it deals with the missing data for the dependent variable in a natural way, enabling us to use all available data in the analysis.

### Sexual and Gender Prejudice

We describe the analysis of the prejudice measures in three subsections. In the first two, we report on the results for the two models of modern LGBT negativity: In the first, we compared modern LGBT negativity across the three time-points in the experimental condition only (Model 1); in the second, we compared differences between the pre- and post-measure of modern LGBT negativity in the experimental vs. control condition (Model 2: only using t0 and t2). In a final subsection, we report the results for old-fashioned prejudice, attitudes toward gender non-conformity, and attitudes toward public displays of affection across the three time-points, again only looking at the experimental group. Descriptive statistics for the different prejudice measures, as a function of condition and time-point, are shown in Table 1.

**Table 1:** Descriptive Statistics Prejudice Measures

	Experimental condition			Control condition	
	t0	t1	t2	t0	t2
	*M* (*SD*)	*M* (*SD*)	*M* (*SD*)	*M* (*SD*)	*M* (*SD*)
Modern LGBT negativity	22.82 (15.74)	19.95 (15.46)	22.29 (16.21)	23.21 (13.22)	26.15 (14.82)
Old-fashioned prejudice	1.96 (0.56)	1.92 (0.64)	1.90 (0.63)	1.92 (0.71)	1.98 (0.79)
Attitudes toward gender non-conformity	2.06 (1.28)	1.98 (1.11)	2.15 (1.22)	2.03 (1.10)	1.96 (1.10)
Attitudes toward public displays of affection	2.60 (1.43)	3.01 (1.44)	2.92 (1.39)	2.32 (1.28)	2.47 (1.33)

Modern LGBT negativity is measured on 100-point scale; other constructs are measured on 7-point scales

Absolute ranges for each measure: modern LGBT negativity (t0: 1.08–61.50. t1: 0.75–64.50. t2: 0.00–64.17). Old-fashioned prejudice (t0: 1.00–3.90. t1: 1.00–3.90. t2: 1.00–4.40). Attitudes toward gender non-conformity (t0: 1.00–5.50. t1: 1.00–5.50. t2: 1.00–5.75). Attitudes toward public displays of affection (t0: 1.00–6.00. t1: 1.00–6.50. t2: 1.00–5.75)

#### Modern LGBT Negativity Across Time in the Experimental Condition (Model 1)

**Fig. 1:**
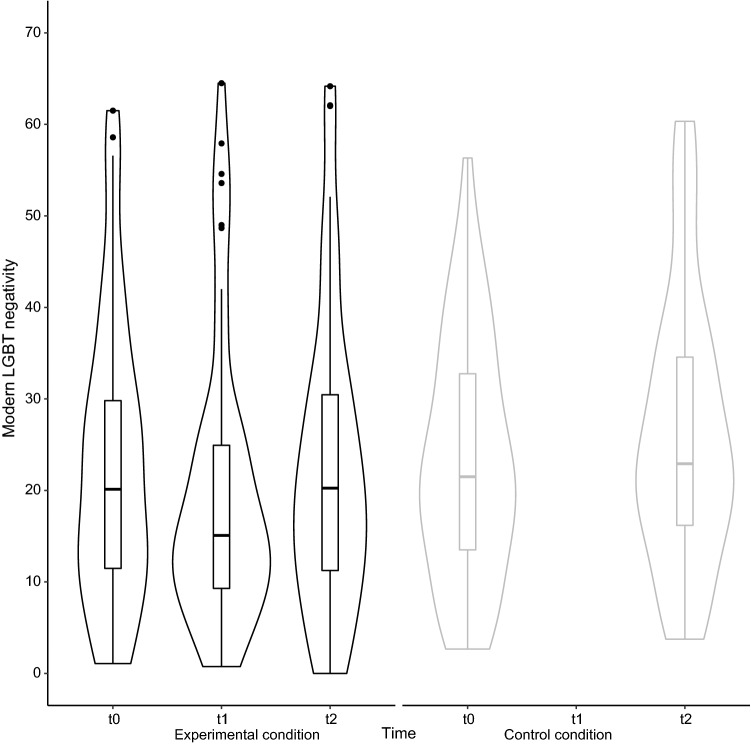
Violin plots on modern LGBT negativity on three different time-points for the intervention group and two different time-points for the control groups

We observed that the changes in modern LGBT negativity scores from pre-intervention (t0) over post-intervention (t1) to follow-up (t2) followed a piecewise linear pattern for most participants. Therefore, to accurately model this pattern, a piecewise representation of time was adopted by creating two dummy variables (Raudenbush & Bryk, 2002): The first dummy modeled the pre- to post-intervention comparison (dummy1: t0 to t1), and the second dummy modeled post-intervention to follow-up comparison (dummy2: t1 to t2). An overview of the estimates for the final model is shown in Table 2 (column “Model 1”). Violin plots displaying means and SD on modern LGBT negativity in the experimental condition are displayed in the left panel of Fig. 1.

**Table 2:** Estimates (and standard errors) for the effects included in the final models for the prediction of modern LGBT negativity

	Model 1	Model 2
*Level 1 fixed effects*		
Intercept	56.48 (6.46)***	56.27 (6.72)***
Dummy1 (t0-t1)	− 3.40 (1.18)**	
Dummy2 (t1-t2)	2.34 (0.80)**	
Dummy3 (t0-t2)		− 0.95 (1.17)
*Level 2 fixed effects*		
Sex (0 = Man)	− 19.44 (3.64)***	− 19.38 (3.79)***
*Level 3 fixed effects*		
Condition (0 = Experimental)		− 20.08 (10.65)
Dummy1*Condition		
Dummy3*Condition		3.93 (1.66)*
Sex*Condition		12.21 (5.85)*†
*Random effects*		
${\sigma }_{\mathrm{error}}^{2}$	17.13	35.80
${\sigma }_{\mathrm{intercept} (\mathrm{student})}^{2}$	147.17	142.90
${\sigma }_{\mathrm{dummy}1 (\mathrm{student})}^{2}$	38.25	
${\rho }_{\mathrm{intercept},\mathrm{dummy}1 (\mathrm{student})}^{2}$	-0.19	

^*^p < .050, **p < .010, ***p < .001

^†^ not-signiicant in bootstrap

Immediately after the intervention (t1), the average participant scored significantly lower on modern LGBT negativity compared to before the intervention (t0; Dummy1: *b* = -3.40, *t* = -2.89, *p* = 0.005). However, at the follow-up measurement (t2), participants showed, on average, higher modern LGBT negativity scores compared to t1 (Dummy2: *b* = 2.34, *t* = 2.94, *p* = 0.005), but still lower—although not significantly so—than before the intervention (*b* = -1.11, *t* = -1.05, *p* = 0.296). Regarding sex, women in general scored (much) lower on modern LGBT negativity than men (Sex: *b* = -19.44, *t* = -5.34, *p* < 0.001). However, the change over time in modern LGBT negativity scores did not differ between men and women (Dummy1*Gender: *b* = 1.72, *t* = 0.67, *p* = 0.507; Dummy2*Gender: *b* = -0.31, *t* = -0.18, *p* = 0.862).

We found large individual variation in the scores of the intercepts (a variance of 147.17) and in the slopes for dummy1 (a variance of 38.25). So, participants not only started out with large differences in modern LGBT negativity scores before the intervention, but they also showed quite diverse immediate reactions to the intervention. Although we found some small violations of the homoscedasticity assumption (i.e., Level 1 residuals having a larger spread at t0 than at t1 and Level 2 residuals for Dummy 1 differing in variance between sexes), the clustered bootstrap analysis confirmed our multilevel results.

Thus, in general, the results showed that the contact intervention had a positive immediate effect in reducing modern LGBT negativity, but no longer-term effect. Moreover, although women scored lower on prejudice than men (which is in line with previous findings; Jäckle & Wenzelburger, 2015), the change in modern LGBT negativity over time in the experimental condition was the same for men and women.

#### Comparing the Intervention Effect Between Conditions (Model 2: t0, t2)

Next, we compared the “longer-term” effect of the intervention on modern LGBT negativity (by comparing pre-intervention t0 with the follow-up measurement t2 between the experimental and the control condition; see Fig. 1). We again also assessed the influence of additional predictors on this effect (i.e., age, sex, and group size). An overview of the estimates for the final model is shown in Table 2 (column “Model 2”). Regarding the longer-term change in modern LGBT negativity due to the intervention, we observed a significant Time by Condition interaction (Dummy3*condition: *b* = 3.93, *t* = 2.37, *p* = 0.020), indicating that the change in modern LGBT negativity between t0 to t2 was different in the experimental condition than in the control condition. In particular, for an average participant in the experimental condition, the intervention did not significantly change modern LGBT negativity (Dummy3: *b* = -0.95, *t* = -0.81, *p* = 0.418). This is what we already noted in the analysis above, where we described Model 1. For an average participant in the control condition, however, modern LGBT negativity scores were 2.98 points higher at t2 than at t0 (*b* = 2.98, *t* = 2.54, *p* = 0.013).

With regard to sex differences, we observed a significant Sex by Condition interaction which indicated that the difference between men and women in modern LGBT negativity at t0 was smaller in the control condition than in the experimental condition. At t0, women in the experimental condition scored, on average, lower on modern LGBT negativity than men in the experimental condition (Sex: *b* = −19.38, *t* = -5.12, *p* < 0.001). In the control condition, however, this difference between women and men at t0 was about 12 units smaller (Sex*condition: *b* = 12.21, *t* = 2.09, *p* = 0.039), with women still scoring lower than men, although not significantly so (*b* = -7.17, *t* = −1.61, *p* = 0.110). However, this interaction effect was non-significant with bootstrapping analyses, decreasing our confidence in the robustness of this finding. Note that this interaction did not change over time (*b* = –3.41, *t* = *−*0.87, *p* = 0.387).

#### Other Forms of Prejudice

We then examined—in the experimental group only—the effect of the intervention on other forms of prejudice, namely old-fashioned prejudice, attitudes toward gender non-conformity, and attitudes toward public displays of affection. In order to assess how participants in the experimental condition responded to the intervention in terms of these different types of prejudice, we again built the multilevel models as described above. However, we found no other variables than time and sex (fixed or random) that were consistently present in the models for the different types of prejudice. Therefore, we decided to adopt the same multilevel model for all four measures (see Table 3). This multilevel model included a piecewise representation of time (Dummy 1 and Dummy 2) and allowed a sex effect on possible changes over time in prejudice.

**Table 3:** Estimates (and standard errors) of the effects in the models for the development of modern and old-fashioned prejudice, and attitudes toward gender non-conformity and public displays of affection in the experimental condition

Parameter	Criterion	Coefficient (SE)
*Level 1 fixed effects*		
Intercept	Modern LGBT negativity	57.88 (6.82)***
	Old-fashioned prejudice	3.00 (0.25)***
	Att. gender non-conformity	4.34 (0.59)***
	Att. public displays of affection	4.71 (0.68)***
Dummy 1	Modern LGBT negativity	− 6.35 (4.57)*
	Old-fashioned prejudice	0.08 (0.23)
	Att. gender non-conformity	− 0.42 (0.43)
	Att. public displays of affection	− 0.25 (0.47)
Dummy 2	Modern LGBT negativity	2.86 (3.08)
	Old-fashioned prejudice	− 0.24 (0.21)
	Att. gender non-conformity	− 0.09 (0.43)
	Att. public displays of affection	0.28 (0.45)
*Level 2 fixed effects*		
Sex	Modern LGBT negativity	− 20.26 (3.86)***
	Old-fashioned prejudice	− 0.61 (0.14)***
	Att. gender non-conformity	− 1.33 (0.34)***
	Att. public displays of affection	− 1.24 (0.39)**
Dummy 1 * Sex	Modern LGBT negativity	1.72 (2.58)
	Old-fashioned prejudice	− 0.07 (0.13)
	Att. gender non-conformity	0.20 (0.24)
	Att. public displays of affection	0.39 (0.27)
Dummy 2 * Sex	Modern LGBT negativity	− 0.31 (1.74)
	Old-fashioned prejudice	0.13 (0.12)
	Att. gender non-conformity	0.15 (0.24)
	Att. public displays of affection	− 0.22 (0.25)

*p < .05, **p < .01, ***p < .001

**Fig. 2:**
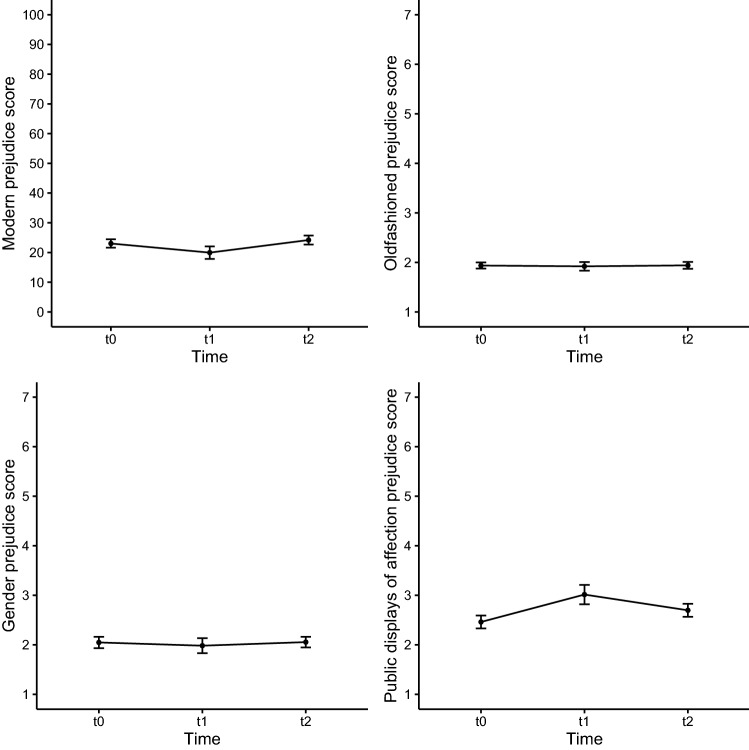
Scores on different prejudice measures on three different time-points for the intervention groups *Note* Error bars represent standard errors

As can be seen in Fig. 2, which displays the change over time for the different forms of prejudice, except for modern LGBT negativity (as described above), prejudice stayed the same before (t0) and after the intervention (t1-t2). Moreover, across all prejudice scales, sex was a significant predictor (see Table 3), with men in general showing higher prejudice than women. Moreover, the sex effect was stable over time (i.e., no interaction between sex and the two dummies for time). Thus, it can be concluded that the intervention had a short-lived effect on modern LGBT negativity, but not on old-fashioned prejudice, attitudes toward gender non-conformity, and attitudes toward public displays of affection.

To examine the possible role of demand characteristics in reporting prejudice, we analyzed the social desirability scale. In general, people scored moderately on social desirability (*M* = 11 on a 20-point scale). More importantly, correlations between social desirability and prejudice were generally low and non-significant. The only exceptions was the correlation between social desirability and old-fashioned prejudice at t1 for participants in the experimental condition, *r*(52) = -0.34, *p* = 0.011. Importantly, social desirability was not significantly correlated with modern LGBT negativity at any time-point (*r*s <|.21|, *p*s > 0.137).

### Evaluation of the Intervention

Because the main goal of this research was to investigate the effectiveness of an existing intervention, we were also interested in how participants responded to the intervention in their self-reported evaluations. Only the data of the 56 participants from the nine groups that received the intervention were included in this analysis. (Note that for five participants from the intervention groups, no data regarding the evaluation of the intervention were collected.) For 14 of these 56 participants, the evaluation scores on t1 were missing.

Participants evaluated the contact intervention quite positively, both directly after the session (t1, *M* = 5.10, *SD* = 0.97 on a 7-point scale; ICC^4^ = 0.20) and in the follow-up test one week later (t2, *M* = 5.46, *SD* = 1.11; ICC = 0.11). A three-level multilevel model (with random slopes and intercepts for subjects and groups) indicated that participants had an even more positive evaluation on the follow-up test than directly after the contact intervention, *t*(41) = 3.44, *p* = 0.001. The positive evaluation was also reflected in how participants graded the contact intervention on a 10-point scale both at t1 (*M* = 8.13, *SD* = 1.00; ICC = 0) and t2 (*M* = 7.71, *SD* = 1.08; ICC = 0). The grade given at the follow-up test was significantly lower, however, than the grade that was given directly following the contact intervention, *t*(55) = -4.19, *p* < 0.001 (tested with the same three-level mixed model as above).

A three-level mixed model analysis also indicated that participants were more inclined to think that the intervention had positively changed their views on LGBT’s on the follow-up test (t2, *M* = 4.39, *SD* = 1.21; ICC = 0.33) compared to directly after the intervention (t1, *M* = 3.61, *SD* = 1.26; ICC = 0.03), *t*(55) = 4.56, *p* = 0.001. This indicated that although the evaluation of the program decreased somewhat over time, the perceived effectiveness of the contact intervention only increased over time.

In line with the aims of the contact intervention, experienced empathy after the intervention was relatively high (*M* = 5.32, *SD* = 0.77; ICC = 0.02), while feelings of unsafety during the intervention were low (*M* = 2.32, *SD* = 0.83; ICC = 0).

Finally, the open-ended questions about the positive and less positive aspects of the contact intervention indicated that participants particularly liked (1) the personal coming-out stories, (2) the open way in which educators responded to questions, and (3) the positive atmosphere educators created in the group. As less positive points, (1) some participants also thought that discussing people’s associations with LGBT was not relevant, (2) some participants would have liked more (scientific) information on LGBT issues, and (3) some participants would have liked more opportunities to be involved in the conversation with the educators.

### Behavior During the Intervention

We explored the correlations between, on the one hand, the percentage of time educators spend talking and participants’ comments, and, on the other hand, the self-report measures of prejudice and the evaluation of the intervention (see Table 4). Responses to the self-report measures were averaged at the group level.

**Table 4:** Correlations between behavior of guest lecturers and participants during intervention and group level self-reported variables

Group level variable	*r*	*p*
*% Of time guest lecturers spent talking*		
t0 Modern LGBT negativity	− .50	.208
t1 Modern LGBT negativity	− .12	.775
t2 Modern LGBT negativity	.08	.856
difference t0-t1 Modern LGBT negativity	− .40	.323
difference t0-t2 Modern LGBT negativity	− .60	.115
difference t1-t2 Modern LGBT negativity	− .31	.463
t1 Evaluation of classroom intervention	.37	.375
t2 Evaluation of classroom intervention	.30	.474
t1 Experienced empathy after intervention	− .07	.867
t1 grade	− .20	.640
t2 grade	− .28	.508
t1 effectiveness of intervention	− .16	.706
t2 effectiveness of intervention	.10	.822
*% Of time participants made comments*		
t0 Modern LGBT negativity	.40	.328
t1 Modern LGBT negativity	− .09	.829
t2 Modern LGBT negativity	− .17	.694
difference t0-t1 Modern LGBT negativity	.45	.263
difference t0-t2 Modern LGBT negativity	.53	.175
difference t1-t2 Modern LGBT negativity	.13	.766
t1 Evaluation of classroom intervention	.61	.112
t2 Evaluation of classroom intervention	.07	.875
t1 Experienced empathy after intervention	.02	.964
t1 grade	.07	.866
t2 grade	.13	.761
t1 effectiveness of intervention	.72	.045
t2 effectiveness of intervention	.45	.258

Boldfaced correlations are significant at *p* < .05

Only the percentage of time participants spent on making comments had a significant positive relationship with the rated effectiveness of the program as measured at t1 (*r*(7) = 0.72, *p* = 0.045, meaning that the more time participants spent making comments, the more they felt the contact intervention had positively changed how they viewed LGBTs. No other significant correlations were observed, which is due to the very low power for this analysis because this analysis was performed at group level (and there were only 9 experimental groups).

## Discussion

In this research, we investigated the effectiveness of an often-used contact intervention in educational settings, aimed at reducing sexual and gender prejudice (Cramwinckel et al., 2018). Although modern LGBT negativity decreased in the experimental groups directly after the intervention, it returned to baseline levels one week after the intervention. Thus, the actual reduction in modern LGBT negativity was short-lived. However, in the control condition, modern LGBT negativity increased over the course of time, even to such an extent that it was higher than in the experimental condition. For old-fashioned prejudice, attitudes toward public displays of affection, and gender identity prejudice, no significant effects were observed. The latter may be partly explained by a possible floor effect because especially old-fashioned prejudice was low in the current population, as anticipated. Moreover, in line with previous work, we observed higher prejudice for men than for women, although the strength of this sex effect was the same regardless of time-point and condition. Finally, the contact intervention was generally positively evaluated; participants graded the intervention highly, were particularly positive about the open atmosphere, open attitude and open way of communication during the intervention, and felt that their attitudes toward LGBTs had become more positive after the intervention (and even more so a week after the intervention).

### Implications

The study has theoretical as well as practical implications. The immediate effect of the intervention on modern LGBT negativity is supportive of contact theory (Allport, 1954; Brown & Hewstone, 2005; Paluck et al., 2021; Pettigrew, 1998; Pettigrew & Tropp, 2006; Smith et al., 2009) even though it was only short-lived. However, measuring longer-term effects (> 1 day) of contact interventions, as we did in the current research, is quite uncommon. A recent meta-analysis (Paluck et al., 2021) demonstrated that out of the 28 experimental studies that used a face-to-face contact intervention, only 8 studies measured outcomes at least one day after the intervention. Furthermore, these longer-term effects were substantially lower than outcomes assessed directly after the intervention. In the current study, we assessed prejudice +—11 days before the intervention, directly after the intervention, and a week after the intervention. Thus, waning effectiveness of interventions over time is unfortunately not uncommon in prejudice reduction research.

One reason why the intervention effect did not last over time may have been that the intervention was too short to create lasting and meaningful bonds (i.e., friendship) between in-group and out-group members. A recent review on the effectiveness of interventions to reduce LGBT prejudice highlights the potential of developing alliances between in-group and out-group members for long-lasting prejudice reduction (Cramwinckel et al., 2018). In the current study, participants had contact with LGBT guest educators for 45–60 min, after which the contact ended. It may be that more intensive contact is necessary to create long-lasting change. For example, White and colleagues (2014) investigated inter-group bias with regard to religion. In this study, Christian and Muslim participants participated in a nine-week virtual intervention where they cooperated with members from other religious groups (experimental condition) or not (control condition). Results demonstrated that inter-group bias was reduced in the experimental conditions, and the effect lasted for at least twelve months after the intervention. Furthermore, reduction was strongest for those participants who had out-group friendships. So perhaps interventions such as the one used in the current research need to be part of a longer-term curriculum, where participants have the opportunity to form meaningful bonds with minority group members.

Further analysis of participants’ evaluations of the intervention, as well behavior during the intervention, provides evidence for the importance of some of the “contact conditions,” that is, the conditions for contact to have an optimal effect in reducing prejudice (Cook, 1985). One condition that has particular relevance for the current intervention was the “acquaintance potential” of the contact situation. As described in the introduction, a core aspect of the current intervention was the “coming-out story” of the educator, which precisely serves the goals of getting to know members of the LGBT community at a very personal level and to increase empathy. It is therefore particularly noteworthy that the coming-out story was the most positively evaluated aspect of the current intervention.

Another aspect of the intervention that was particularly well evaluated concerns the open and positive atmosphere during the meeting. Moreover, the more time participants spent on discussing comments, the more they thought that the intervention was effective. However, these findings might have two relevant implications: First, they are in line with another contact condition, namely a positive and collaborative atmosphere. The second implication is that although the active role of the educator is a crucial aspect of the intervention, this should perhaps not go at the expense of the opportunity for participants to actively engage themselves. Indeed, these exploratory results suggest that the more participants were talking, the more effective the intervention was in positively changing participants’ views on LGBT people. This is in line with other recent work showing positive effects of voicing one’s opinions to peers. Wu and Paluck (2020), for example, observed that workers’ objective productivity, as well as their job satisfaction and empowerment, increased after they engaged in interactive sessions where they could voice their opinions to their supervisors and coworkers (vs. control conditions where supervisors gave short lectures on expected performance and company goals). Thus, the current results suggest that a positive and open atmosphere where participants are actively engaged through discussions and other activities is a core aspect of a successful contact intervention. However, these exploratory findings should be followed up by more rigorous testing within larger samples.

A result that needs to be discussed in this context concerns the finding that participants had even more positive evaluations of the contact intervention on the follow-up measure one week after the intervention than directly after the intervention. There are at least two explanations for this. The first is that despite that the initial evaluation of the contact intervention was also quite positive, the contact intervention may have been somewhat confrontational at the same time. For example, playing the “statement game” where a coming-out experience was simulated by sharing (sensitive) personal information, may have caused some feelings of discomfort or stress. These feelings may have dissipated one week later, resulting in an even more positive evaluation of the contact intervention. The second possible reason for why the evaluation was even more positive one week later is that in the meanwhile participants may have discussed the contact intervention with each other, which may in turn have further strengthened their already positive attitude.

The methodology of the current study offers some important contributions for literature and practice on LGBT prejudice reduction. First, we assessed the influence of the intervention on several forms of LGBT prejudice (Cramwinckel et al., 2018). From a theoretical and practical point of view, the best intervention would reduce the multiple facets of sexual and gender prejudice simultaneously. However, as is clear from the results, we only observed significant (but short-lived) reductions of modern LGBT negativity (adapted from Morrison & Morrison, 2003). Explicit (“old-fashioned”) prejudice, attitudes toward public displays of affection, and attitudes toward gender non-conformity did not change as a result of the intervention. Important to note is that these forms of prejudice were low in this sample (i.e., the observed pattern could be indicative of floor effects for these forms of prejudice). Therefore, it is not possible to draw firm conclusions about the effect of this intervention on these other forms of prejudice, as they were barely present in the current sample.

Second, this study shows the benefits of using an experimental design with intervention and control groups. Such an approach is relatively rare in research on sexual and gender diversity contact interventions (Cramwinckel et al., 2018). The results demonstrated very different effects on modern LGBT negativity over time in the experimental and the control groups. In the experimental groups, modern LGBT negativity at t0 and t2 was roughly similar (with a short-lived reduction in prejudice at t1). In the control conditions, modern LGBT negativity was higher at t2 than at t0. Comparing the experimental condition with the control condition demonstrated that the contact intervention can perhaps even be considered effective in the long run, because the increase in prejudice in the control condition did not appear in the experimental condition, which suggests that the intervention weakened an increase in prejudice that might otherwise have also been present in the experimental condition.

### Limitations

One important limitation that needs to be addressed is the small sample size with respect to between-group comparisons in the current study, given that we have only 18 groups (but about 50 participants per condition). This is unfortunate, because larger samples would have enabled us to draw firmer conclusions, for example about the correlations between behavioral and self-reported data. However, we assessed three within-participants measurements of our key variables on LGBT prejudice, thereby increasing power on the measures that were of most interest to the current work.

Although the current results suggest a modestly positive direct influence of the contact intervention in reducing sexual and gender prejudice, a puzzling aspect of the results concerns the increase in modern LGBT negativity in the control condition. How can this be explained? We offer two explanations. A first possible explanation relates to the “history” threat to internal validity in longitudinal research designs (Judd et al., 1991). It may have been the case that something happened outside the laboratory (e.g., in the “real world”) between the pretest and the follow-up measure that influenced prejudice scores in the control group. Although it is hard to exclude this possibility completely, we have no clear indication that such an event with regard to this topic occurred during this period, making this explanation somewhat less likely. A second possible explanation for the observed increase in prejudice over time relates to the repeated exposure to prejudice-related statements through the repeated prejudice measurements. Paradoxically, making stereotype- and prejudice-related judgments also activates, reinforces, and in turn strengthens these cognitive and affective associations (Payne et al., 2017), which has been a noted limitation to diversity and anti-bias training programs (Dobbin & Kalev, 2018). These paradoxical effects may be particularly likely for more modern forms of prejudice because people do not recognize it as such (Cramwinckel et al., 2018). That is, while more old-fashioned, blatant forms of prejudice are easily recognized and condemned, modern prejudice tends to build-up every time people encounter it (Krolikowsky et al., 2016). On top of that, such effects may be further strengthened by awareness about the goal of the project (i.e., prejudice reduction), which could have led to reactance among at least some participants. When participants experience too much external pressure during prejudice interventions, this may have the paradoxical effect of increasing, rather than reducing, prejudice (Legault et al., 2011). Together, these findings underline how difficult it is to obtain sustainable prejudice reductions through bias intervention programs, and that even merely repeatedly assessing prejudicial attitudes (i.e., without an intervention), can have negative side-effects.

Another limitation of the current study might be the possible operation of demand characteristics. This issue becomes especially relevant when considering that the most clear effect of the intervention took place shortly after the contact situation. In the current study, the participants were generally aware that we were interested in the effectiveness of the intervention (i.e., there was no deception) which may have led participants to report lower prejudice directly after the intervention than before. However, in the instructions for the questionnaires, it was stressed that “there were no right or wrong answers” and that we were “only interested in their personal opinion.” Moreover, analyses of the Social Desirability Scale showed that although social desirability was related to the expression of lower levels of old-fashioned prejudice (as may be expected to some extent), social desirability was completely unrelated to the expression of modern prejudice at any point in time. Thus, we are confident that demand characteristics did not drive the reduction in modern prejudice at t1 for participants in the experimental condition.

Finally, although more active engagement during the intervention related to a more positive evaluation of the program afterward, we should be cautious in drawing too firm conclusions regarding the causality of this effect. It is possible that people who had a priori positive impressions about the program were also more engaged during the session and still quite positive about the program afterward.

### Suggestions for Further Research

One important venue for future research concerns testing the generalizability of our findings to other contexts and populations (Bartos et al., 2014). Although the current contact intervention has been commonly used, and although we used trained educators in the current study, other educational settings (such as high schools) might differ in several ways from the current context. In particular, the current sample contained relatively small groups, with highly educated—and mostly female—participants. Sex and education are factors known to be associated with prejudice (as was the case in our research), with higher educated people and women generally scoring lower on prejudice (Cramwinckel et al., 2018; Jäckle & Wenzelburger, 2015). This is also evident from the absolute scores on the prejudice measures in the current study, which were relatively low.

Given these sample characteristics, it is noteworthy that a recent study on a similar intervention program in the Netherlands targeting younger students from lower educational levels found a small increase in prejudice, especially among boys (Kroneman et al., 2019). This latter finding is in line with other research suggesting that high inter-group anxiety and threat can hinder the potential positive effects of contact (Pettigrew, 1998) or that contact can even have a “hardening” effect in some populations (Martin et al., 2007).

At the same time, one might argue that at least under some conditions, there is more room for improvement among participants with moderate or higher baseline levels of prejudice, which would increase the potential impact of an intervention. Indeed, there is research showing that the impact of interventions is the highest for people who have most to gain (Bradshaw et al., 2015). Thus, an important venue for future research is to test the current intervention in other contexts and in other (more prejudiced) populations.

In the current study, we examined the effectiveness of the current contact intervention in a holistic manner, that is, we measured prejudice toward LGBTs generally, and not to specific subgroups (e.g., people who identify as lesbian vs. transgender). This leaves open the possibility that specific attitudes toward different subgroups may differ, also as a result of the intervention (Herek, 2002; Norton & Herek, 2013). However, the aim of this specific intervention program was to target sexual and gender prejudice in a broad sense. Partly as a reflection of this, the intervention class was delivered by educators with different sexual orientations and gender identities. For these reasons, as well as practical constraints, we adapted an existing scale for modern homonegativity (Morrison & Morrison, 2003) and adapted it to cover prejudice toward different sexual orientations and gender identities. However, future research should more systematically examine whether the current intervention reduces sexual and gender prejudice indeed in a more general manner, or whether it is particularly effective in reducing specific forms, and whether the group membership of the educator plays a role in this.

Another consequence of our “holistic” approach was that we were not able to test the effectiveness of specific ingredients of the intervention (e.g., coming-out story vs. statement game). Although we know which aspects of the intervention are generally more positively evaluated, we have less certainty about their actual effectiveness in reducing prejudice. Interventions such as these are costly in terms of time, money, and personnel. If effectiveness is not as high as intended, measures should be taken to increase the effectiveness and/or investigate alternative interventions with potentially a higher impact. Scientific research can help in this endeavor by disentangling effective elements (e.g., in the laboratory) and building an intervention that consists of only effective elements. Thus, an important goal of future research would be to study more in depth the different ingredients of interventions, and how they relate to specific forms of prejudice, with the ultimate aim to combine the most effective ingredients in the most effective intervention.

Based on the literature (e.g., Cramwinckel et al., 2018; Felten et al., 2015), as well as the results of the current study, one element with high potential to reduce modern LGBT negativity seems to be sharing a personal coming-out story. A next step in examining this further would be to test several versions of coming-out stories, as well as the different methods to share them (e.g., face-to-face, via video, written text, etc.) to assess which types of stories and which methods of sharing would be most effective. If, for example, a face-to-face situation is not necessary to increase perspective-taking and empathy, then relatively inexpensive interventions such as video-messages or virtual reality methods may also prove to be effective (e.g., Vang & Fox, 2014). However, recent literature suggests that perspective-taking should happen in an interactive context where people can really learn and interact, which was precisely the aim with the current intervention (Eyal et al., 2018).

### Conclusion

In the current research, we employed a commonly used intervention method by professional guest educators, which contributed to high external validity and applied importance. Furthermore, we used a rigorous methodological design with pre-, post- and follow-up measures, video recordings of actual behavior, random allocation to groups, and assessment of several aspects of prejudice. Participants responded positively to the intervention. The contact intervention had a small and short-lived positive effect on reducing modern LGBT negativity. However, some care should be in place before generalizing these findings directly to applied settings, due to potential differences in sample and setting (e.g., group size, education level, sex composition, etc.). Thus, although there is still ample room for improvement, we conclude that using the current intervention is probably better than not intervening at all.

## Supplementary Information

Below is the link to the electronic supplementary material.

Supplementary file1 (DOCX 42 KB)

## Data Availability

Stimulus materials, raw data, syntaxes, and other study materials are stored on the Open Science Framework and are available via osf.io/s9zwg.

## Footnotes

1 In the current work, we discuss sexual orientation and gender identity prejudice as a single class of prejudice. Despite the important differences between sub-classes (e.g., prejudice toward lesbians vs. gay men; Herek, 2002; Norton & Herek, 2013), there are theoretical as well as practical reasons for discussing them together here. First, sexual orientation prejudice is strongly related to, and may even be rooted in, the rejection of gender diversity (Cramwinckel et al., 2018). Second, the contact intervention that we investigated in the current work was developed to reduce prejudice related to both sexual and gender diversity, and the educators providing the intervention were members of sexual and/or gender minorities.

2 Religion was only assessed at t1 for participants in the experimental condition.

3 COC Mid-Netherlands is sub-organization of COC, which is the largest Dutch organization that strives for equal rights for, and emancipation of, sexual and gender minorities (see www.coc.nl). The program is currently taught hundreds of times each year, especially to Dutch high school students, thereby reaching thousands of students every year. More specifically, the intervention was implemented 304 times in 2017, 403 times in 2016, and 313 times in 2015. Most of these implementations took place in high schools, reaching adolescents in the ages 13–16 years. However, implementation in other educational settings (e.g., first-year university students; teachers; youth workers; students in vocational tracks; primary school students) has become increasingly common in recent years.

4 The intra-class correlation (ICC) quantifies how much variance in the scores can be attributed to differences between groups (instead of differences between subjects within groups). Note that ICC equals zero when groups do not differ on average.

## Code Availability

Stimulus materials, raw data, syntaxes, and other study materials are stored on the Open Science Framework and are available via osf.io/s9zwg.
