# Supplementary material for: Assessing the Effects of a Real-Life Contact Intervention on Prejudice Toward LGBT People
Source: Arch Sex Behav. 2021 Sep 9;50(7):3035–51. doi: 10.1007/s10508-021-02046-0 (PMC8563548; doi:10.1007/s10508-021-02046-0)
Supplement: Supplementary file 1 — Supplementary file1 (DOCX 42 KB) [file 10508_2021_2046_MOESM1_ESM.docx]

| **Table S1**  *Overview of all measures in study* | | | | | | |
| --- | --- | --- | --- | --- | --- | --- |
|  | **Construct** | **Time points** | **Conditions** | **Measurement** | **English items** | **Dutch items** |
| **Background Variables** | | | | | | |
|  | Gender | t0 | all | categorical | **What is your biological sex?** | **Wat is je geslacht?** |
|  | Age | t0 | all | open ended | **What is your age?** | **Wat is je leeftijd?** |
|  | Sexual Orientation | t0 | all | categorical | **What is your sexual orientation?** | **Wat is je seksuele orientatie?** |
|  | LGBT contact | t0 | all | open ended | **How many lesbians, gays, transgenders and or bisexuals do you know in person?** | **Hoeveel lesbiennes, homo's, transgenders en/of biseksuelen ken jij persoonlijk?** |
|  | LGBT friends | t0 | all | open ended | **How many of these lesbians, gays, transgenders and/or bisexuals do you consider to be (good) friends?** | **Hoeveel van deze lesbiennes, homo's, transgenders en/of biseksuelen beschouw jij als goede vrienden?** |
| **Sexual Orientation and Gender Identity Prejudice Measures** | | | | | | |
|  | **oldfashioned prejudice** |  |  |  |  |  |
|  | (ATG-R-S5; ATL-R-S5; Herek, 1997) | t0, t1, t2 | all | 7-point Likert Scale ranging from 1(disagree strongly) - 7 (agree strongly) | **I think male homosexuals are disgusting** | **Ik vind homoseksuele mannen vies** |
|  |  |  |  |  | **Male homosexuality is a perversion** | **Mannelijke homoseksualiteit is een perversie** |
|  |  |  |  |  | **Male homosexuality is a natural expression of sexuality in men (reversed)** | **Mannelijke homoseksualiteit is een natuurlijke vorm van seksualiteit bij mannen (reversed)** |
|  |  |  |  |  | **Sex between two men is just plain wrong** | **Seks tussen twee mannen is gewoon verkeerd** |
|  |  |  |  |  | **Male homosexuality is merely a different kind of lifestyle that should not be condemned (reversed)** | **Mannelijke homoseksualiteit is simpelweg een ander soort levensstijl waar niet over geoordeeld zou moeten worden (reversed)** |
|  |  |  |  |  | **Lesbians just can't fit into our society** | **Lesbiennes passen niet in onze samenleving** |
|  |  |  |  |  | **State laws against private sexual behavior between consenting adult women should be abolished (reversed)** | **Wetgeving die het seksuele privéleven van twee instemmende volwassen vrouwen inperkt, zou verbannen moeten worden (reversed)** |
|  |  |  |  |  | **Female homosexuality is a sin** | **Vrouwelijke homoseksualiteit is een zonde** |
|  |  |  |  |  | **Female homosexuality in itself is no problem unless society makes it a problem (reversed)** | **Lesbisch-zijn is op zichzelf geen probleem, behalve als de samenleving er een probleem van maakt (reversed)** |
|  |  |  |  |  | **Lesbians are sick** | **Lesbiennes zijn ziek** |
|  | **modern prejudice** |  |  |  |  |  |
|  | MHS; Morrison & Morrison (2002) | t0, t1, t2 | all | continuous slider bar ranging from 0 (disagree strongly) - 100 (agree strongly) | **LGBT's use their sexual orientation so that they can obtain special privileges** | **Lesbiennes, homoseksuelen, biseksuelen en transgenders gebruiken hun seksuele oriëntatie zodat ze speciale privileges kunnen krijgen** |
|  | items adapted such that 'gay men' is replaced with Lesbians, Gays, Bisexuals and Transgendered people | | | | **LGBT's seem to focus on ways in which they differ from heterosexuals, and ignore the ways in which they are the same** | **Lesbiennes, homoseksuelen, biseksuelen en transgenders focussen zich op de dingen waarop ze verschillen van heteroseksuelen en negeren de overeenkomsten** |
|  |  |  |  |  | **LGBT's do not have all the rights they need (reversed)** | **Lesbiennes, homoseksuelen, biseksuelen en transgenders hebben niet alle rechten die ze nodig hebben** |
|  |  |  |  |  | **The notion of universities providing students with undergraduate degrees in Gay and Lesbian studies is ridiculous** | **Het is belachelijk dat universiteiten vakken aanbieden over homoseksualiteit** |
|  |  |  |  |  | **Celebrations such as "Gay pride day" are ridiculous because they assume that an individual's sexual orientation should constitute a source of pride** | **Feestdagen zoals "Gay Pride Day" zijn belachelijk, omdat ze ervan uitgaan dat iemands seksuele voorkeur iets is waar iemand trots op kan zijn** |
|  |  |  |  |  | **LGBT's still need to protest for equal rights (reversed)** | **Lesbiennes, homoseksuelen, biseksuelen en transgenders moeten nog steeds opkomen voor hun rechten (reversed)** |
|  |  |  |  |  | **LGBT's should stop shoving their lifestyle down other people's throats** | **Lesbiennes, homoseksuelen, biseksuelen en transgenders moeten stoppen met hun levenstijl opdringen aan anderen** |
|  |  |  |  |  | **If LGBT's want to be treated like everyone else, then they need to stop making such a fuss about their sexuality/culture** | **Als lesbiennes, homoseksuelen, biseksuelen en trangenders hetzelfde als anderen behandeld willen worden, moeten ze niet zo'n punt maken van hun seksualiteit/cultuur** |
|  |  |  |  |  | **LGBT's who are "out of the closet" should be admired for their courage (reversed)** | **Lesbiennes, homoseksuelen, biseksuelen en transgenders die "uit de kast" zijn zouden voor hun moed bewonderd moeten worden (reversed)** |
|  |  |  |  |  | **LGBT's should stop complaining about the way they are treated in society and simply get on with their lives** | **Lesbiennes, homoseksuelen, biseksuelen en transgenders zouden moeten stoppen met protesteren over hoe ze in deze samenleving behandeld worden en gewoon hun leven leiden** |
|  |  |  |  |  | **In today's tough economic times, tax dollars shouldn't be used to support gay and lesbian organisations** | **In deze zware economische tijden, zou de regering geen belastinggeld moeten gebruiken om homo en lesbische organisaties te steunen** |
|  |  |  |  |  | **LGBT's have become far too confrontational in their demand for equal rights** | **Lesbiennes, homoseksuelen, biseksuelen en transgenders zijn veel te confronterend geworden wat betreft hun eisen voor gelijke rechten** |
|  | **Attitudes towards public displays of affection** |  |  |  |  |  |
|  | (Kuypers, 2015) | t0, t1, t2 | all | 7-point Likert Scale ranging from 1(disagree strongly) - 7 (agree strongly) | **I find it offensive when two men kiss in public** | **Ik vind het aanstootgevend als twee mannen in het openbaar zoenen** |
|  |  |  |  |  | **I find it offensive when two women kiss in public** | **Ik vind het aanstootgevend als twee vrouwen in het openbaar zoenen** |
|  |  |  |  |  | **I find it offensive when a man and a woman kiss in public** | **Ik vind het aanstootgevend als een man en een vrouw in het openbaar zoenen** |
|  |  |  |  |  | **I find it less offensive to see a man and woman holding hands in public, then to see two men holding hands** | **Als ik een man en een vrouw hand in hand zie lopen in het openbaar heb ik daar minder moeite mee dan wanneer ik twee mannen hand in hand zie lopen** |
|  | **Attitudes towards gender non-conformity** |  |  |  |  |  |
|  | (Kuypers, 2015) | t0, t1, t2 | all | 7-point Likert Scale ranging from 1(disagree strongly) - 7 (agree strongly) | **I do not feel comfortable around masculine women** | **Ik voel mij niet op mijn gemak bij vrouwen die er mannelijk uitzien** |
|  |  |  |  |  | **I do not feel comfortable around femine men** | **Ik voel mij niet op mij gemak bij mannen die er vrouwelijk uitzien** |
|  |  |  |  |  | **Feminine men are asking for trouble** | **Als een man zich vrouwelijk gedraagt, dan vraagt hij om problemen** |
|  |  |  |  |  | **Masculine women are asking for trouble** | **Als een vrouw zich mannelijk gedraagt, dan vraagt zij om problemen** |
| **Evaluation of the Intervention** | | | | | | |
|  | Effectiveness of the intervention | t1, t2 | experimental | 7-point Likert Scale ranging from 1(disagree strongly) - 7 (agree strongly) | **This education class changed my views on lesbians, gays, bisexuals and transgenders for the better** | **Deze voorlichting heeft mijn kijk op lesbiennes, homoseksuelen, biseksuelen en transgenders in positieve zin veranderd** |
|  | **Evaluation of the classroom intervention** | t1, t2 | experimental | 7-point Likert Scale ranging from 1(disagree strongly) - 7 (agree strongly) | **I thought the education class was informative** | **Ik vond de voorlichting informatief** |
|  |  | t1, t2 | experimental | 7-point Likert Scale ranging from 1(disagree strongly) - 7 (agree strongly) | **I thought the education class was useless** | **ik vond de voorlichting zinloos** |
|  |  | t1, t2 | experimental | 7-point Likert Scale ranging from 1(disagree strongly) - 7 (agree strongly) | **I thought the education class was useful** | **Ik vond de voorlichting zinvol** |
|  | Most positive aspects | t1 | experimental | open ended | **Which aspects of the education class made the most positive impression on you and why?** | **Welke aspecten van de voorlichting hebben de meest positieve indruk op je gemaakt en waarom?** |
|  | Least positive aspects | t1 | experimental | open ended | **Which aspects of the education class made the least positive impression on you and why?** | **Welke aspecten van de voorlichtingen hebben de minst positieve indruk op je gemaakt en waarom?** |
|  | Grade education class | t1, t2 | experimental | Grade (0 - 10) | **How do you grade the education class? 1 is the lowest grade, and 10 the highest grade.** | **Welk cijfer geef je deze voorlichting? 0 is het laagste cijfer, en 10 het hoogste cijfer** |
|  | Grade guest lecturers | t1 | experimental | Grade (0 - 10) | **How do you grade the guest lecturers? 1 is the lowest grade, and 10 the highest grade.** | **Welk cijfer geef je deze voorlichters? 0 is het laagste cijfer, en 10 het hoogste cijfer** |
|  | **Experienced empathy after intervention** | t1 | experimental | 7-point Likert Scale ranging from 1(disagree strongly) - 7 (agree strongly) | **I thought the guest lecturer's coming out story was inspiring** | **Ik vond het coming out verhaal van de voorlichter inspirerend** |
|  |  | t1 | experimental |  | **I felt moved by the eduction class** | **De voorlichting ontroerde mij** |
|  |  | t1 | experimental |  | **I empathized with the guest lecturers' stories** | **Ik voelde mee met het verhaal van de voorlichters** |
|  |  | t1 | experimental |  | **I felt uncomfortable during the education class** | **Ik voelde mij ongemakkelijk tijdens de voorlichting** |
|  |  | t1 | experimental |  | **I sympathized with the guest lecturers** | **Ik voel sympathie voor de voorlichters** |
|  |  | t1 | experimental |  | **It angers me to realize that the guest lecturers have been judged on their sexual orientation** | **Het maakt mij boos om te beseffen dat de voorlichters wel eens negatief beoordeeld zijn op hun seksuele oriëntatie.** |
|  |  | t1 | experimental |  | **I could empathize with the personal stories of the guest lecturers** | **Ik kon me inleven in het verhaal van de voorlichter(s)** |
|  |  | t1 | experimental |  | **I had trouble imaginging putting myself in the guest lecturers' shoes** | **Ik had moeite om me in de schoenen te plaatsen van de voorlichter(s)** |
|  | **Feelings of unsafety** | t1 | experimental | 7-point Likert Scale ranging from 1(disagree strongly) - 7 (agree strongly) | **During the education class, I was worried that people would think I was prejudiced** | **Ik maakte me zorgen tijdens de voorlichting dat mensen zouden denken dat ik vooroordelen had** |
|  |  | t1 | experimental |  | **During the education class, I feared that my opinions would be critized** | **Ik was bang dat mijn mening tijdens de voorlichting bekritiseerd zou worden** |
|  |  | t1 | experimental |  | **I felt uncomfortable when I learned about the guest lecturers' section orientation** | **Ik voelde me ongemakkelijk toen ik hoorde wat de seksuele oriëntatie van de voorlichter(s) was** |
|  |  | t1 | experimental |  | **I though it was exciting to be in the education class** | **Ik vond het spannend om de voorlichting bij te wonen** |
|  |  | t1 | experimental |  | **During the education class, I felt free to openly express my opinions** | **Ik durfde tijdens de voorlichting openlijk mijn mening te verkondigen** |
|  |  | t1 | experimental |  | **I felt inhibited during the education class** | **Ik voelde me tijdens de voorlichting geremd** |
|  |  | t1 | experimental |  | **We respected each other's opinions during the education class** | **Tijdens de voorlichting respecteerden we elkaars mening** |
|  |  | t1 | experimental |  | **I felt free to give my opinion during the education class** | **Ik durfde tijdens de voorlichting mijn mening te geven** |
|  |  | t1 | experimental |  | **I dared to disagree with the guest lecturers** | **Ik durfde de voorlichter(s) tegen te spreken** |
| **Additional measures** | | | | | | |
|  | **Etnicity** | t0 | all | categorical | What is your etnicity? | Wat is je etniciteit? |
|  | **Religiosity** | t2 | all | categorical (yes; no) | Are you religious? | Ben je religieus? |
|  | **Religion** | t2 | all | open ended | What is your religious affiliation? | Welk geloof hang je aan? |
|  | **Importance of Religion** | t2 | all | 7-point Likert scale ranging from 1 (not important at all) to 7 (very important) | How important is religion to you? | Hoe belangrijk is religie voor je? |
|  | **Remarks** | t0, t1 | all | open ended | Did you notice something particular about this study and/or do you have any questions or remarks for the experimenters? | Is je iets opgevallen over dit onderzoek en/of heb je nog vragen of opmerkingen voor de onderzoekers? |
|  | **Attitudes towards equal rights** |  |  |  |  |  |
|  | (Kuypers, 2015) | t0, t1, t2 | all | 7-point Likert Scale ranging from 1(disagree strongly) - 7 (agree strongly) | Gaymarriage should be abolished | Het homohuwelijk dient te worden afgeschaft |
|  |  |  |  |  | Gay couples should have the same rights as heterosexual couples | Homoseksuele paren moeten dezelfde rechten hebben als heteroseksuele paren |
|  | **Empathy** |  |  |  |  |  |
|  | Toronto Empathy Questionnaire (TEQ; Spreng, McKinnon, Mar, & Levine, 2009) | t0 | all | 7-point Likert Scale ranging from 1(disagree strongly) - 7 (agree strongly) | When someone else is feeling excited, I tend to get excited too | Als iemand anders enthousiast is, ben ik geneigd ook enthousiast te worden |
|  |  |  |  |  | Other people’s misfortunes do not disturb me a great deal (reversed) | Andermans tegenslagen doen mij niet zoveel (reversed) |
|  |  |  |  |  | It upsets me to see someone being treated disrespectfully | Ik word verdrietig wanneer iemand anders respectloos wordt behandeld |
|  |  |  |  |  | I remain unaffected when someone close to me is happy (reversed) | Het doet mij niet zoveel als iemand om mij heen blij is (reversed) |
|  |  |  |  |  | I enjoy making other people feel better | Ik vind het fijn om andere mensen zich beter te laten voelen |
|  |  |  |  |  | I have tender, concerned feelings for people less fortunate than me | Ik heb tedere, bezorgde gevoelens voor mensen die minder gelukkig zijn dan ik |
|  |  |  |  |  | When a friend starts to talk about his\her problems, I try to steer the conversation towards something else (reversed) | Als een vriend of vriendin over zijn/haar gevoelens begint, probeer ik van onderwerp te veranderen (reversed) |
|  |  |  |  |  | I can tell when others are sad even when they do not say anything | Ik heb het door wanneer iemand zich rot voelt zelfs wanneer diegene dat niet zegt |
|  |  |  |  |  | I find that I am “in tune” with other people’s moods | Ik pik de stemming van anderen makkelijk op |
|  |  |  |  |  | I do not feel sympathy for people who cause their own serious illnesses(reversed) | Ik voel geen sympathie voor mensen die hun eigen ziekte veroorzaken (reversed) |
|  |  |  |  |  | I become irritated when someone cries (reversed) | Ik word geïrriteerd wanneer iemand huilt (reversed) |
|  |  |  |  |  | I am not really interested in how other people feel (reversed) | Ik ben niet echt geïnteresseerd in hoe andere mensen zich voelen (reversed) |
|  |  |  |  |  | I get a strong urge to help when I see someone who is upset | Ik krijg een sterke behoefte om te helpen wanneer iemand overstuur is |
|  |  |  |  |  | When I see someone being treated unfairly, I do not feel very much pity for them(reversed) | Wanneer ik zie dat iemand unfair wordt behandeld, voel ik soms weinig medelijden met diegene (reversed) |
|  |  |  |  |  | I find it silly for people to cry out of happiness (reversed) | Ik vind het gek als mensen huilen van geluk (reversed) |
|  |  |  |  |  | When I see someone being taken advantage of, I feel kind of protective towards him\her | Wanneer ik zie dat er misbruik van iemand wordt gemaakt, heb ik de behoefte hem/haar te beschermen |
|  | **Behavioral Inhibition / Behavioral Activation** | |  |  |  |  |
|  | BIS/BAS (Carver & White, 1994). | t0 | all | 7-point Likert Scale ranging from 1(disagree strongly) - 7 (agree strongly) | If I think something unpleasant is going to happen I usually get pretty "worked up." | Ik raak enigzins gestrest als ik denk dat er iets vervelends gaat gebeuren |
|  |  |  |  |  | I worry about making mistakes | Ik pieker wel eens over het maken van fouten |
|  |  |  |  |  | Criticism or scolding hurts me quite a bit | Kritiek of uitbranders raken mij behoorlijk |
|  |  |  |  |  | I feel pretty worried or upset when I think or know somebody is angry at me | Ik voel me bezorgd of overstuur als ik denk of weet dat iemand boos op me is |
|  |  |  |  |  | Even if something bad is about to happen to me, I rarely experience fear or nervousness | Ik voel zelden angst of zenuwen, zelfs als me iets vervelends te wachten staat |
|  |  |  |  |  | I feel worried when I think I have done poorly at something important | Ik maak me zorgen als ik denk dat ik slecht heb gepresteerd |
|  |  |  |  |  | I have very few fears compared to my friends | Ik ervaar weinig angsten vergeleken met mijn vrienden |
|  |  |  |  |  | When I get something I want, I feel excited and energized | Als ik krijg wat ik wil, voel ik me opgewonden en energiek |
|  |  |  |  |  | When I'm doing well at something I love to keep at it | Als ik iets goed doe, wil ik er graag mee doorgaan |
|  |  |  |  |  | When good things happen to me, it affects me strongly | Als ik iets leuks meemaak heeft dat duidelijk invloed op me |
|  |  |  |  |  | It would excite me to win a contest | Als ik een wedstrijd zou winnen, zou ik erg enthousiast zijn |
|  |  |  |  |  | When I see an opportunity for something I like I get excited right away | Als ik ergens een buitenkansje zie dan word ik meteen enthousiast |
|  |  |  |  |  | I go out of my way to get things I want | Als ik iets wil, zal ik er gewoonlijk alles aan doen om dit te krijgen |
|  |  |  |  |  | When I want something I usually go all-out to get it | Ik zal mijn uiterste best doen om de dingen te krijgen die ik wil |
|  |  |  |  |  | If I see a chance to get something I want I move on it right away | Als ik de kans zie iets te krijgen wat ik wil, zal ik die kans grijpen |
|  |  |  |  |  | When I go after something I use a "no holds barred" approach | Als ik iets van plan ben dan laat ik mij door niets weerhouden |
|  |  |  |  |  | I will often do things for no other reason than that they might be fun | Vaak doe ik dingen alleen voor de lol |
|  |  |  |  |  | I crave excitement and new sensations | Ik verlang naar spanning en sensaties |
|  |  |  |  |  | I'm always willing to try something new if I think it will be fun | Ik ben altijd bereid iets nieuws te proberen als ik denk dat het leuk zal zijn |
|  |  |  |  |  | I often act on the spur of the moment | Ik doe vaak dingen in een vlaag van opwelling |
|  |  |  |  |  |  |  |
|  | **Social Desirability** |  |  |  |  |  |
|  | (M-C 1; M-C 2; Strahan & Gerbasi, 1972) | t0 | all | binary (true; false) | I'm always willing to admit it when I make a mistake | Ik ben altijd bereid toe te geven dat ik een fout heb gemaakt |
|  |  |  |  |  | I always try to practice what I preach | Ik probeer altijd te doen wat ik zeg |
|  |  |  |  |  | I never resent being asked to return a favor | Ik vind het nooit vervelend om iemand een wederdienst te bewijzen |
|  |  |  |  |  | I have never been irked when people expressed ideas very different from my own | Het heeft me nog nooit geërgerd als mensen een total andere mening hebben dan ik |
|  |  |  |  |  | I have never deliberately said something that hurt someone's feelings. | Ik heb nog nooit opzettelijk iets gezegd om iemand te kwetsen |
|  |  |  |  |  | I like to gossip at times | Ik vind het af en toe leuk om te roddelen |
|  |  |  |  |  | There have been occasions when I took advantage of someone | Ik heb wel eens misbruik van iemand anders gemaakt |
|  |  |  |  |  | I sometimes try to get even rather than forgive and forget | Soms wil ik liever wraak nemen dan iemand vergeven |
|  |  |  |  |  | At times I have really insisted on having things my own way | Op sommige momenten heb erg hard mijn zin doorgedrukt |
|  |  |  |  |  | There have been occasions when I felt like smashing things | Er zijn gevallen geweest waarbij ik zin had om dingen kapot te maken |
|  |  |  |  |  | I never hesitate to go out of my way to help someone in trouble | Ik twijfel nooit om er alles aan te doen om iemand te helpen |
|  |  |  |  |  | I have never intensely disliked anyone | Ik heb nog nooit een intense hekel aan iemand gehad |
|  |  |  |  |  | When I don't know something I don't at all mind admitting it. | Als ik iets niet weet, heb ik er geen enkel probleem mee om dat toe te geven |
|  |  |  |  |  | I would never think of letting someone else be punished for my wrong doings. | Het zou niet in me opkomen om iemand anders te laten straffen voor mijn fouten |
|  |  |  |  |  | I sometimes feel resentful when I don't get my way | Soms voel ik me wraakzuchtig als ik mijn zin niet krijg |
|  |  |  |  |  | There have been times when I felt like rebelling against people in authority even though I knew they were right | Ik heb wel eens zin gehad om in opstand te komen tegen autoriteiten zelfs wanneer ik wist dat ze gelijk hadden |
|  |  |  |  |  | I can remember "playing sick" to get out of something | Ik heb wel eens gedaan alsof ik ziek was om onder iets uit te komen |
|  |  |  |  |  | There have been times when I was quite jealous of the good fortune of others | Ik ben wel eens behoorlijk jaloers geweest op het geluk van anderen |
|  |  |  |  |  | I am sometimes irritated by people who ask favors of me | Ik raak soms geïrriteerd als iemand me om een gunst vraagt |
|  |  |  |  |  | I am always courteous, even to people who are disagreeable | Ik ben altijd beleefd, zelfs tegen mensen die onvriendelijk zijn |
|  | **Collective Self-esteem** |  |  |  |  |  |
|  | (CSE, Luhtanen & Crocker, 1992. membership self-esteem subscale, private collective self-esteem subscale, public collective self-esteem subscale) | t0, t1, t2 | all | 7-point Likert Scale ranging from 1(disagree strongly) - 7 (agree strongly) | I often regret that I belong to my tutorgroup | Ik vind het jammer dat ik bij mijn tutoraatgroep hoor |
|  |  |  |  |  | Overall, my tutorgroup is considered good by others [t1: by the guest lecturers] | Mijn tutoraatgroep zou waarschijnlijk positief worden beoordeeld door anderen |
|  | "social groups" replaced by "my tutorgroup" in all items | |  |  | In general, I'm glad that I belong to my tutorgroup | Ik ben blij dat ik bij mijn tutoraatgroep hoor |
|  |  |  |  |  | Most people [t1: the guest lecturers] consider my tutorgroup, on the average, to be more ineffective than other tutorgroups | De meeste mensen zouden mijn tutoraatgroep waarschijnlijk minder effectief vinden dan andere groepen |
|  |  |  |  |  | Overall, I often feel that my tutorgroup is not worthwile | Ik heb het gevoel dat mijn tutoraatgroep niet de moeite waard is |
|  |  |  |  |  | In general, others [t1: the guest lecturers] respect my tutorgroup | Andere mensen zouden waarschijnlijk respect hebben voor mijn tutoraatgroep |
|  |  |  |  |  | I feel good about my tutorgroup | Ik voel me goed over mijn tutoraatgroep |
|  |  |  |  |  | In general, others [t1: the guest lecturers] think that my tutorgroup is unworthy | Andere mensen zouden mijn tutoraatgroep waarschijnlijk onwaardig vinden |
|  |  |  |  |  | I am a worthy member of my tutorgroup | Ik ben een waardevol lid van mijn tutoraatgroep |
|  |  |  |  |  | I feel I don't have much to offer to my tutorgroup | Ik heb het gevoel dat ik niet veel kan bijdragen aan mijn tutoraatgroep |
|  |  |  |  |  | I am a cooperative participant in my tutorgroup | Ik ben een coöperatief lid van mijn tutoraatgroep |
|  |  |  |  |  | I often feel I'm a useless member of my tutorgroup | Ik voel me een waardeloos lid van mijn tutoraatgroep |
|  | **Entitativity** |  |  |  |  |  |
|  | Entitativity measure (Postmes, Brooke, & Jetten, 2008, cf. Lakens & Stel, 2011) | t0, t1, t2 | all | 7-point Likert Scale ranging from 1(disagree strongly) - 7 (agree strongly) | I feel my tutorgroup is a unit | Mijn tutoraatgroep vormt een eenheid |
|  |  |  |  |  | I think my tutorgroup can act in unison | Mijn tutoraatgroep gedraagt zich als één |
|  |  |  |  |  |  |  |
|  |  |  |  |  | I experience a feeling of togetherness in my tutorgroup | Er is veel saamhorigheid in mijn tutoraatgroep |
|  |  |  |  |  | I feel my tutorgroup is as one | Mijn tutoraatgroep is als één |
|  | **Familiarity with tutorgroup** |  |  |  |  |  |
|  |  | t0, t1 | all | 7-point Likert Scale ranging from 1(disagree strongly) - 7 (agree strongly) | I know the others in my tutorgroup very well | Ik ken de anderen in mijn tutoraatgroep erg goed |
|  |  |  |  |  | I consider the others in my tutorgroup to be good friends of mine | Ik beschouw de anderen in mijn tutoraatgroep als goede vrienden |
|  |  |  |  |  |  |  |
|  | **Additional evaluation of intervention items** | |  |  |  |  |
|  |  | t1 | experimental | 7-point Likert Scale ranging from 1(disagree strongly) - 7 (agree strongly) | I thought the education class was fun | Ik vond de voorlichting leuk |
|  |  | t1 | experimental |  | I thought the education class was boring | Ik vond de voorlichting saai |
|  |  | t1 | experimental |  | I liked the guest lecturers | Ik vond de voorlichter(s) aardig |
|  |  | t1 | experimental |  | I thought the guest lecturers were pleasant | Ik vond de voorlichter(s) sympathiek |
|  |  | t2 | experimental |  | The education class taught me about LGBT issues | Na de voorlichting is mij meer duidelijk geworden over aspecten van homoseksualiteit |
|  |  | t2 | experimental |  | I think it's easier to "come out" after the education class | Ik denk dat het makkelijker is na de voorlichting om uit de kast te komen |
|  |  | t2 | experimental |  | I remember a lot from the education class | Ik kan mij veel herinneren over de voorlichting |
|  |  | t2 | experimental |  | I thought the education class was informative | Ik vond de voorlichting leerzaam |
|  |  | t2 | experimental |  | After the education class, I felt comfortable discussing LGBT issues | Ik voel mij gemakkelijk om te praten over LHBT na de voorlichting |
|  |  | t2 | experimental |  | I think it's important that these education classes are taught | Ik vind het belangrijk dat deze voorlichtingen worden gegeven |
|  |  | t2 | experimental |  | It's easier to empathize with LGBT issues after participating in the education class | Na de voorlichting kan ik mij beter inleven in LHBT |
|  | Earlier experience | t1 | experimental | categorical (yes; no; I don't know) | Do you have earlier experience with this kind of education classes? | Heb je al eens een soortgelijke voorlichting bijgewoond? |
|  | Number of guest lecturers | t1 | experimental | categorical (1; 2; 3) | How many guest lecturers were present? | Hoeveel voorlichters waren aanwezig bij de voorlichting? |
|  | **Group Atmosphere** |  |  |  |  |  |
|  |  | t1 | experimental | 7-point Likert Scale ranging from 1(disagree strongly) - 7 (agree strongly) | The vibe in the group was pleasant during the education class | Tijdens de voorlichting was de sfeer in de groep fijn |
|  |  | t1, t2 | experimental |  | I would think it's weird if someone in my tutorgroup was LGBT | Als iiemand in mijn tutoraatgroep LHBT* is, zou ik dat raar vinden |
|  |  | t1 | experimental |  | I think that at least one of my groupmembers is LGBT | Ik denk dat minimaal één van mijn groepsgenoten LHBT* is |
|  |  | t1 | experimental |  | Is someone in your tutorgroup "out of the closet"? | Is er iemand in jouw tutoraatgroup uit de kast? |
|  | **Behavioral Support** |  |  |  |  |  |
|  | (Fingerhut, 2011) | t1 | experimental | categorical (yes; no; I don't know) | |  |
|  |  |  |  |  |  |  |
|  | "Could you indicate whether or not you participated or performed these activities in the past month?" | t1 | experimental | categorical (yes, performed/participated; no, did not perform/participate; I don't know) | Attending a gay rights event | Bezoeken van een evenement gericht op het bevorderen van rechten van seksuele minderheden (lesbiennes, homoseksuelen, biseksuelen en transgenders) |
|  |  |  |  |  | Donating time to organizations promoting LGBT rights | Vrijwilliger zijn voor een organisatie die opkomt voor de rechten van seksuele minderheden |
|  |  |  |  |  | donating money to organizations promoting LGBT rights | Geld doneren aan een organisatie die opkomt voor de rechten van seksuele minderheden |
|  |  |  |  |  | Sigining a petition for equal rights for sexual minorities | Het ondertekenen van een petitie voor gelijke rechten voor seksuele minderheden |
|  |  |  |  |  | Participating in discussions promoting LGBT rights | Deelnemen aan discussies om rechten voor seksuele minderheden te verbeteren |
|  |  |  |  |  | Initiating discussions promoting LGBT rights | Het initiëren van discussies om rechten voor seksuele minderheden te verbeteren |
|  | **Additional self-developed prejudice items** | t0, t1, t2 | all | 7-point Likert Scale ranging from 1(disagree strongly) - 7 (agree strongly) | It would bother me if gays or lesbians would think that I am prejudiced | Ik zou het vervelend vinden als homo's of lesbiennes denken dat ik vooroordelen over hen heb |
|  |  |  |  |  | I feel that lesbians and gays always think that heterosexuals are prejudiced | Ik heb het idee dat lesbiennes en homo's altijd denken dat heteroseksuelen vooroordelen over hen hebben |
|  |  |  |  |  | I do not think that it's necessary to have affirmative action programs to protect the rights of sexual minorities (lesbians, gays, bisexuals and transgenders) | Ik vind het niet nodig dat er speciale programma's zijn die opkomen voor seksuele minderheden (lesbiennes, homo's, biseksuelen en transgenders) |
|  |  |  |  |  | Sexual minorities unfairly often feel disadvantaged | Seksuele minderheden voelen zich vaak onterecht benadeeld |
|  |  |  |  |  | There is hardly any prejudice against sexual minorities (lesbians, gays, bisexuals and transgenders) | Er zijn nauwelijks meer vooroordelen tegenover seksuele minderheden (lesbiennes, homo's, biseksuelen en transgenders) |
|  |  |  |  |  | I see no differences between sexual minorities (lesbians, gays, bisexuals and transgenders) and heterosexuals | Ik zie geen verschil tussen seksuele minderheden (zoals homo's en lesbiennes) en heteroseksuelen |
|  |  |  |  |  | Lesbians, gays, transgenders and bisexuals are equal to heterosexuals | Lesbiennes, homo's, trangsenders en biseksuelen zijn gelijk aan hetero's |
|  |  |  |  |  | I think it's important that people realize that I am not prejudiced against gays | Ik vind het belangrijk dat mensen merken dat ik geen vooroordelen heb over homo's |
| *Note*. Items in boldface are included in the scales and reported in the main text | | | |  |  |  |
|  |  |  |  |  |  |  |
|  |  |  |  |  |  |  |
